# Ectopic Osteogenesis of Macroscopic Tissue Constructs Assembled from Human Mesenchymal Stem Cell-Laden Microcarriers through *In Vitro* Perfusion Culture

**DOI:** 10.1371/journal.pone.0109214

**Published:** 2014-10-02

**Authors:** Maiqin Chen, Min Zhou, Zhaoyang Ye, Yan Zhou, Wen-Song Tan

**Affiliations:** State Key Laboratory of Bioreactor Engineering, School of Bioengineering, East China University of Science and Technology, Shanghai, China; National Institutes of Health, United States of America

## Abstract

We had previously demonstrated the feasibility of preparing a centimeter-sized bone tissue construct by following a modular approach. In the present study, the objectives were to evaluate osteogenesis and tissue formation of human amniotic mesenchymal stem cells-laden CultiSpher S microcarriers during *in vitro* perfusion culture and after subcutaneous implantation. Microtissues were prepared in dynamic culture using spinner flasks in 28 days. In comparison with 1-week perfusion culture, microtissues became more obviously fused, demonstrating significantly higher cellularity, metabolic activity, ALP activity and calcium content while maintaining cell viability after 2-week perfusion. After subcutaneous implantation in nude mice for 6 and 12 weeks, all explants showed tight contexture, suggesting profound tissue remodeling *in vivo*. In addition, 12-week implantation resulted in slightly better tissue properties. However, *in vitro* perfusion culture time exerted great influence on the properties of corresponding explants. Degradation of microcarriers was more pronounced in the explants of 2-week perfused macrotissues compared to those of 1-week perfusion and directly implanted microtissues. Moreover, more blood vessel infiltration and bone matrix deposition with homogeneous spatial distribution were found in the explants of 2-week perfused macrotissues. Taken together, *in vitro* perfusion culture time is critical in engineering bone tissue replacements using such a modular approach, which holds great promise for bone regeneration.

## Introduction

Tissue engineering aims at fabricating viable tissue replacements to repair and restore structure and function of damaged tissues by employing scaffolds and cells [1]. In the conventional methodology, cells are seeded onto prefabricated three-dimensional (3D) scaffolds, followed by cell proliferation, deposition of extracellular matrix (ECM) and remodeling of microenvironments to form organotypic structure. However, one major issue of this procedure is that cells preferentially proliferate in peripheral regions (within 200–250 µm) of constructs due to a limit in mass transport [2]–[4]. Dimensions of tissue engineered constructs reported so far generally fall in a maximum of several millimeters [2], [3], [5]. Hence, there is an urgent need of developing new strategies to fabricate large tissues to meet clinical needs, such as bone repair.

Different routes have been exploited to address this issue. One is to cultivate constructs in dynamic fluid flow in bioreactors to promote mass transport. Beneficial effects of perfusion on cell survival, proliferation and tissue formation have been reported [6], [7]. Nevertheless, dynamic culture in bioreactors does not change the mechanism of mass transport inside constructs with high cellularity, which is essentially passive diffusion [8], [9]. Another potential strategy is to build in a functional vascular network in *in vitro* engineered tissues, which is expected to quickly establish anastomosis with hosts once implanted [10].

Recently, a modular approach has emerged [11]. In this approach, microscopic tissues (microtissues) as building blocks are prepared and then assembled into macroscopic tissues (macrotissues) following a “bottom-up” mechanism [11]. Microtissues with diverse forms have been reported, including cellular aggregates, cellular sheets and cell-laden microgels/microcarriers [12]–[15]. Due to the small sizes of these microtissues (generally within several hundred µm), high cell density with great viability can be easily achieved. In addition, these fabrication processes are adaptable for large scale production [15], [16]. To assemble microtissues into macrotissues, several different strategies are explored such as random packing, stacking of cellular sheets and directed assembly [11]. For example, Du et al. encapsulated cells in polyethylene glycol (PEG) microgels, which were submerged in hydrophobic mineral oil to assemble into a tubular-like structure through hydrophobic effects [17]. Takeuchi et al. exploited cell-laden gelatin gel beads, which were randomly packed into a doll-shaped macrotissue in a mold [18]. Palmiero et al. dynamically seeded bovine fibroblasts onto porous microcarriers and then loaded into a disc-shaped chamber (1 cm in diameter, 1 mm in thickness), followed by perfusion culture to induce tissue assembling and maturation [19]. Moreover, engineering vascularized tissues can be conveniently realized by using this modular approach [20].

**Figure 1:**
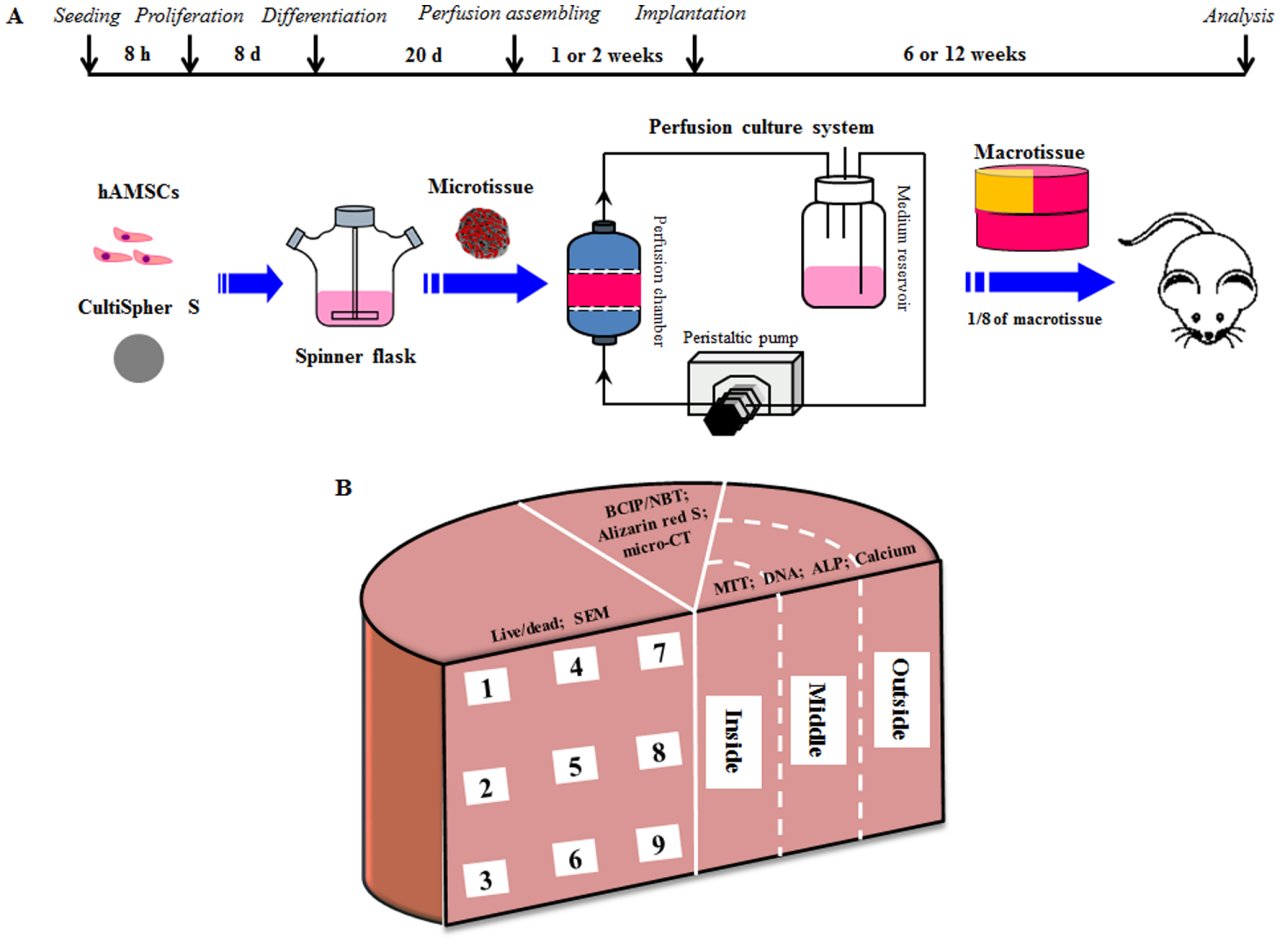
Schematic illustration of experimental design. (**A**) The time table of the experimental process was described. hAMSCs were dynamically seeded onto CultiSpher S microcarriers within 8 h, cultured in growth medium for 8 d and osteogenically differentiated for 20 d in spinner flask. Then, cell-laden microcarriers were loaded into perfusion chamber for assembling into macrotissue for 1 or 2 weeks, followed by implantation into nude mice for 6 or 12 weeks. (**B**) The assembled macrotissues were sampled from different regions for assays.

In an endeavor to fabricate large sized tissue replacements, we applied the modular approach to engineer a centimeter-sized bone tissue assembled from human amniotic mesenchymal stem cells (hAMSCs)-laden microcarriers in perfusion culture in a previous report [21]. Cells were dynamically seeded onto CultiSpher S microcarriers in spinner flasks and allowed to proliferate and differentiate. In a perfusion culture system, these modular bone-like tissues were successfully assembled into a cylindrical construct with a dimension of 2 cm×1 cm (diameter×height) within 7 days. In this process, perfusion culture is expected to provide intensified mass transport during assembling initially and exert mechanical stimulation on cells [6], [22]. However, we previously found that culture of human fibroblasts (HFs)-laden Cytopore-2 microcarriers in a perfusion chamber for 15 days led to significant cell death in a cylindrical macrotissue (length×diameter: 1.5 cm×2.0 cm), suggesting that an optimal *in vitro* culture time should be considered in order to ensure good quality of tissues [23]. Hence, the objective of the present study was to investigate the effects of *in vitro* perfusion culture time on the properties of tissue constructs assembled from hAMSCs-laden CultiSpher S microcarriers. In addition, whether the *in vitro* perfusion culture time would affect the *in vivo* maturation of these macrotissues was also tested. So far, no studies have concerned the *in vivo* performance of macrotissues fabricated via a modular approach. Specifically, in the present study, perfusion culture of microtissues for assembling was performed for 1 or 2 weeks and the assembled macrotissues were then implanted in nude mice subcutaneously for 6 or 12 weeks as schematically illustrated in Fig. 1A.

## Materials and Methods

### Ethics Statement

Human term placentae were acquired from healthy women with written consent and the protocol of this study had been approved by the ethics committee at East China University of Science and Technology. All animal experiments were performed at Shanghai Laboratory Animal Center and the protocol was approved by the institutional animal care and use committee of Shanghai Laboratory Animal Center. All surgery was performed under sodium pentobarbital anesthesia and all efforts were made to minimize suffering.

### Isolation and culture of hAMSCs

hAMSCs were isolated as previously described [21]. Briefly, the amnion was separated from chorionic, rinsed with phosphate-buffered saline (PBS) buffer and cut into small pieces. Tissue fragments were treated with trypsin-EDTA solution (Gibco) followed by 1 mg/mL collagenase I (Gibco) in Dulbecco's modified Eagle's medium (DMEM, Gibco). Erythrocytes were lysed with NH_4_Ac and cells were plated at a density of 1×10^6^ cells/dish in 10-cm dishes in growth medium consisted of α-MEM (Gibco) supplemented with 10% fetal bovine serum (FBS, Hyclone, USA) at 37°C in a humidified atmosphere of 5% CO_2_. Once confluent, cells were either frozen down or sub-cultured at 1∶4 dilution.

### Fabrication of microtissues

Microtissues were fabricated as described in our previous report [21]. CultiSpher S (Sigma) are macroporous porcine gelatin microcarriers with a diameter of 130∼380 mm and pore size of 10 mm. Microcarriers were rehydrated in Ca^2+^ and Mg^2+^ free PBS, autoclaved and rinsed once with PBS and twice with growth medium. Microcarriers were loaded at 2 mg/mL in 250-mL siliconized spinner flasks (Corning) in growth medium supplemented with 0.05 mg/ml of ascorbic acid-2-phosphate (Sigma) and 5×10^−5^ M of mercaptoethanol (Sigma). Cells were seeded at 5×10^4^ cells/mL using an intermittent stirring regime (3 min agitation at 30 rpm followed by 30 min settling, which was repeated for a total of 8 h). Culture was agitated at 50 rpm and maintained in growth medium for 8 days before medium was replaced with osteogenic medium consisting of DMEM supplemented with 10% FBS, 100 nM dexamethasone (Sigma), 10 mM sodium-β-glycerophosphate (Sigma), 0.1 mg/mL ascorbic acid-2-phosphate and 5×10^−5^ M of mercaptoethanol. Culture lasted for another 20 days and medium change was performed every 2 days.

### Microtissue assembling in perfusion culture

The setup of perfusion culture system was illustrated in Fig. S1. It was composed of three functional parts: cylindrical perfusion chamber (2 cm in diameter), peristaltic pump (Masterflex L/S, Cole Parmer, USA) and medium reservoir. For perfusion culture, 120 mL of microtissue suspension directly taken from spinner flasks was loaded into each perfusion chamber and microtissues were let settled freely. Osteogenic medium was continuously perfused at 1 mL/min for 1 or 2 weeks and refreshed every 2 days.

### Subcutaneous implantation in nude mice

At the end of perfusion culture, assembled macrotissues were retrieved and cut into 8 equal block wedges (Fig. 1A). On the back of each male BALB/c nude mouse aged 6–8 weeks, two subcutaneous pockets were created and one tissue block was implanted in each pocket. For each experimental group (i.e., macrotissues perfused for either 1 or 2 weeks), 12 blocks in total were subcutaneously implanted in nude mice. As a control, 15 mL of microtissue suspension was taken without perfusion assembling, which contained an equal amount of microtissues as 1/8 block of a macrotissue, and implanted in each pocket after decanting the medium. Explants were retrieved after 6 or 12 weeks.

### Live/dead assay

Tissue samples (microtissues, macrotissues or explants) were rinsed with PBS and then incubated with 2 mM calcein-AM (CAM, Sigma), 2 mM propidium iodide (PI, Sigma) and 2 mM 4,6-diamino-2-phenyl indole (DAPI, Sigma) in PBS for 30 min at 37°C. After incubation, samples were rinsed with PBS and examined under confocal laser-scanning microscope (TCS SP5, Leica).

### MTT assay

Samples were treated with 2 mL of growth medium supplemented with 200 µL of 3-(4,5-dimethylthiazolyl-2)-2,5-diphenyltetrazolium bromide (MTT, 5 mg/mL in PBS, Sigma) for 4 h at 37°C in a fully humidified atmosphere of 5% CO_2_ in dark. After that, samples were rinsed twice with PBS and 2 mL of dimethyl sulfoxide was added to extract formazan crystals. Absorbance (optical density, OD) at 570 nm of the extractant was measured on DU 730 UV/Vis spectrophotometer (Beckman Coulter). OD values were normalized to dry weights of respective tissue samples (mg^−1^).

### DNA assay

Tissue samples were treated with papain solution (125 µg/mL papain (Sigma), 5 mM L-cysteine, 100 mM Na_2_HPO_4_, 5 mM EDTA, and pH 6.2) at 60°C overnight. DNA content was measured with Hoeschst 33258 dye (Sigma) using a fluorometer (DQ300, Hoefer). Calf thymus DNA (Invitrogen) was used as standards. DNA contents were normalized to dry weights of respective samples (µg/mg).

### Scanning electron microscope (SEM)

Samples were rinsed with PBS and fixed with 2.5% glutaraldehyde overnight at 4°C. Following PBS wash, samples were treated with 1% osmium tetroxide and 1% tannic acid at 4°C. After dehydration in a graded series of ethanol solutions, samples were dried in a critical point drier and sputtered with gold. SEM images were acquired on a SEM (S-3400N, Hitachi) in conjunction with an energy-dispersive X-ray spectrometer (EDX, Apollo X, EDAX Inc.) at an accelerating voltage of 15 kV. For microtissues, the elemental composition was analyzed with EDX mode.

### Analysis of alkaline phosphatase (ALP) activity

BCIP/NBT alkaline phosphatase color development kit (Beyotime, China) was used to stain samples according to manufacturer's instruction. ALP activity was quantified using alkaline phosphatase assay kit (Nanjing Jiancheng Biological Engineering Research Institute, China) according to the manufacturer's instruction. Samples were rinsed with PBS and treated with 0.2% Triton X-100. The supernatants of cell lysates were incubated at 37°C for 15 min with 10 mM *p*-nitrophenylphosphate in 0.35 M 2-amino-2-methyl-1-propanol (pH 10.4) containing 2 mM Mg^2+^. NaOH solution (0.2 M) was added to stop the reaction. Absorbance at 520 nm was measured on the UV/Visible spectrophotometer. A standard curve was established using a series of phenol solutions. One unit of ALP activity was defined as the production of 1 nmol phenol in 15 min and values were expressed as unit/mg dry weight of respective sample.

### Alizarin red S staining and quantification of calcium content

Samples were rinsed with PBS, fixed in 95% ethanol and stained with 1% w/v Alizarin red S (Sigma). Calcium content in samples was quantified using a calcium-methyl thymol blue method (Nanjing Jiancheng Biological Engineering Research Institute, China). Each sample was treated with 500 mL of 0.1 M HCl at 4°C overnight. 50 mL of the solution was transferred to a test tube containing 1 mL of methyl thymol blue solution and 1 mL of alkaline solution and incubated for 5 min. Absorbance at 610 nm was measured on the UV/Visible spectrophotometer. A standard curve was made with calcium standard solutions between 0 and 2.5 mmol/L. Calcium contents were normalized to dry weights of respective samples (mmol/mg).

### Micro-CT analysis

Samples were fixed in 4% paraformaldehyde and visualized on a micro-CT system (μCT-80, Scanco Medical, Switzerland). Settings were used as follows: pixel matrix, 1024×1024; voxel size, 36 µm; slice thickness, 1000 µm. Three dimensional images were reconstructed from the scans with software package. All reconstructions were obtained using the same standardized threshold.

### Histological staining

Tissue samples were rinsed with PBS, fixed in 4% paraformaldehyde, dehydrated in a graded series of ethanol solutions, embedded in paraffin and sectioned (5 µm) on a microtome (RM2235, Leica). Sections were stained with hematoxylin and eosin (H&E) and Masson's trichrome stain. For immunohistochemical staining, sample sections were incubated with primary antibodies against CD31 (1∶200; Rabbit polyclonal antibody against mouse, CD31, Abcam), followed by horseradish peroxidase (HRP)-conjugated anti-rabbit or anti-mouse secondary antibody (1∶200 in PBS containing 1% BSA, Santa Cruz) and color development with diaminobenzidine tetrahydrochloride (Santa Cruz). Nuclei were counterstained with hematoxylin. Images were taken on a phase contrast microscopy (Eclipse TS100, Nikon). To quantify the positive staining of CD31, for each sample, 3 sections were stained and for each section, 3 images were taken for analysis. Image J software was applied to determine the mean vessel density by calculating positively stained vessel area per unit area. Artifacts of “false” positive staining for microcarriers were manually excluded during the calculation.

### Statistical analysis

Values were presented as mean ± standard deviation. Numerical data were analyzed statistically using the Student's t-test and p-values of <0.05 were considered significant.

## Results

### Fabrication of bone-like microtissues using hAMSCs and microcarriers

**Figure 2:**
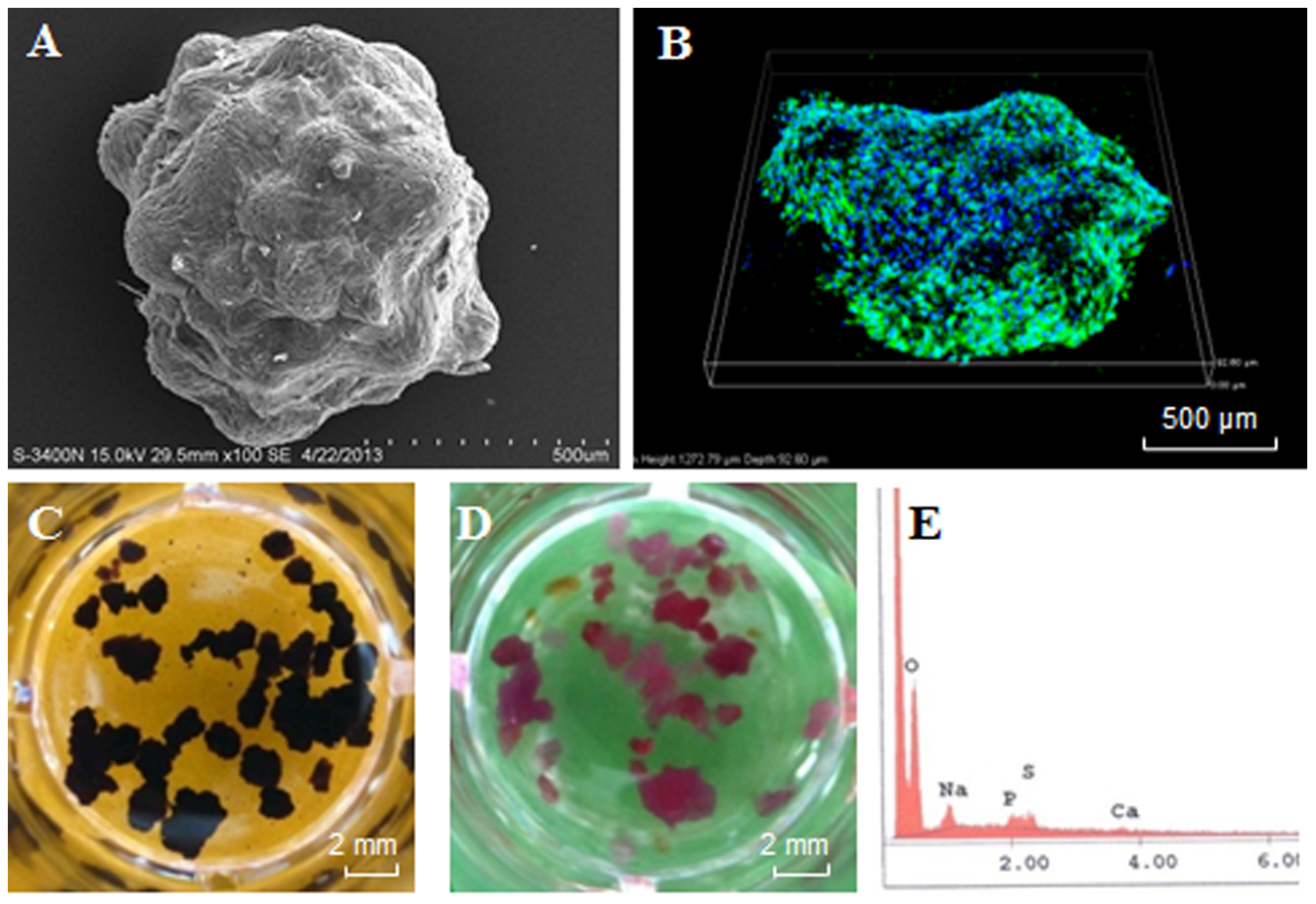
Characterization of bone microtissues. Cell-laden microcarriers formed aggregates in culture, termed as microtissues, and subjected to SEM observation (**A**), live/dead staining (**B**), BCIP/NBT staining for ALP activity (**C**), Alizarin Red S staining for minerals (**D**) and EDX analysis (**E**).

Microtissues were fabricated by culturing hAMSCs on CultiSpher S microcarriers in spinner flasks. Cells were allowed to proliferate for 8 days in growth medium and then osteogenically induced for 20 days (Fig. 1A). Within 28 days, cells proliferated and covered the surface of microcarriers (Fig. 2A). These cell-laden microcarriers tended to aggregate into spheroids of 700∼800 µm in diameter. Live/dead staining showed that hAMSCs remained viable in microtissues (Fig. 2B). ALP activity was confirmed with BCIP/NBT staining (Fig. 2C) and a significant amount of minerals was deposited by cells based on Alizarin red S staining and EDX analysis (Fig. 2D,E).

### Assembling of microtissues into macrotissues in perfusion culture

**Figure 3:**
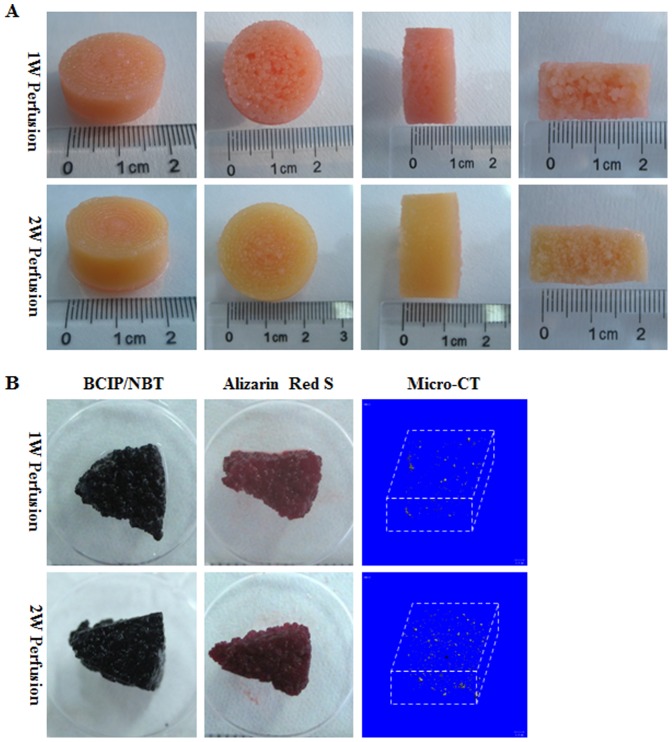
Gross view and osteogenesis of macrotissues. Microtissues were assembled into macrotissues for 1 week (1W Perfusion) or 2 weeks (2W Perfusion) in perfusion culture. (**A**) Cylindrical macrotissues with dimensions of centimeters were prepared. (**B**) Macrotissues were cut into small blocks and subjected to BCIP/NBT staining for ALP activity, Alizarin Red S staining for minerals and micro-CT analysis.

As shown in Fig. 3A, cylindrical macrotissues were prepared with a dimension of ∼2.4 cm×∼1.2 cm (diameter×height) after both 1 and 2 weeks of perfusion culture in osteogenic medium and these macrotissues demonstrated an integral structure without obvious defects regardless of perfusion culture time. However, the macrotissue perfused for 1 week had a more rough cross-section compared to that perfused for 2 weeks. Both macrotissues maintained bone-like tissue characteristics with high ALP activity, calcium deposition and formation of minerals as evidenced by BCIP/NBT staining, Alizarin red S staining and micro-CT analysis, respectively (Fig. 3B).

**Figure 4:**
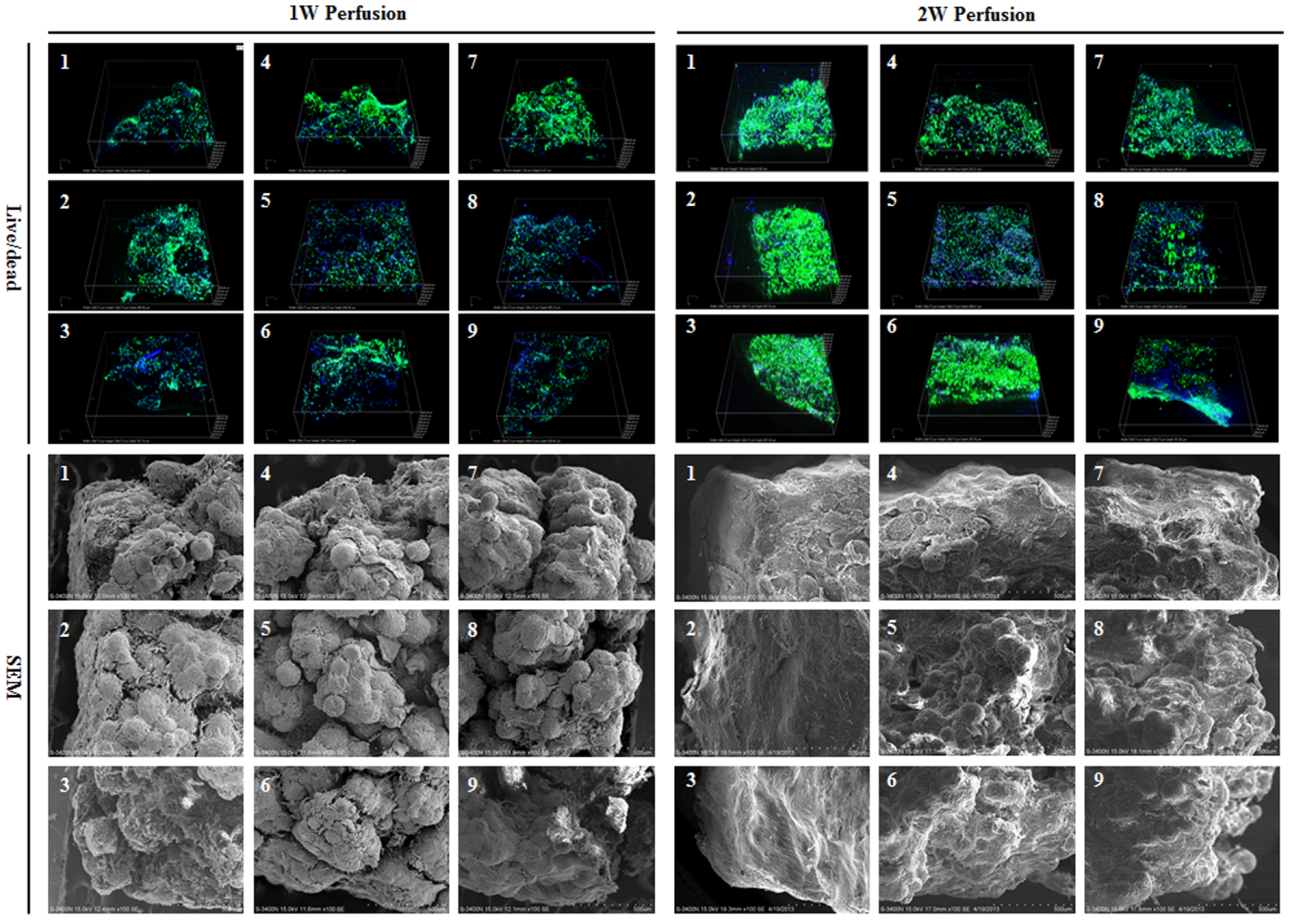
Cell viability and microstructure of macrotissues. Nine different regions of macrotissues were sampled as defined in Fig. 1B. Live/dead staining was performed to determine cell viability and SEM was applied to examine the microstructural features inside macrotissues.

Macrotissues were sampled in 9 different regions as illustrated in Fig. 1B for live/dead assay and SEM examination (Fig. 4). Cells remained viable in all regions in both macrotissues. However, cell density was slightly higher in the macrotissue perfused for 2 weeks, especially in the outer regions (regions “1–3” and “6”). Based on SEM observation, inside the macrotissue perfused for 1 week, all microcarriers were covered with cells and big clumps of cell-laden microcarriers could be clearly seen in interior regions (regions “5–8”), suggesting incomplete fusion between microtissues. In contrast, the boundary of microtissues became invisible in the macrotissue perfused for 2 weeks and almost all cell-laden microcarriers fused together.

**Figure 5:**
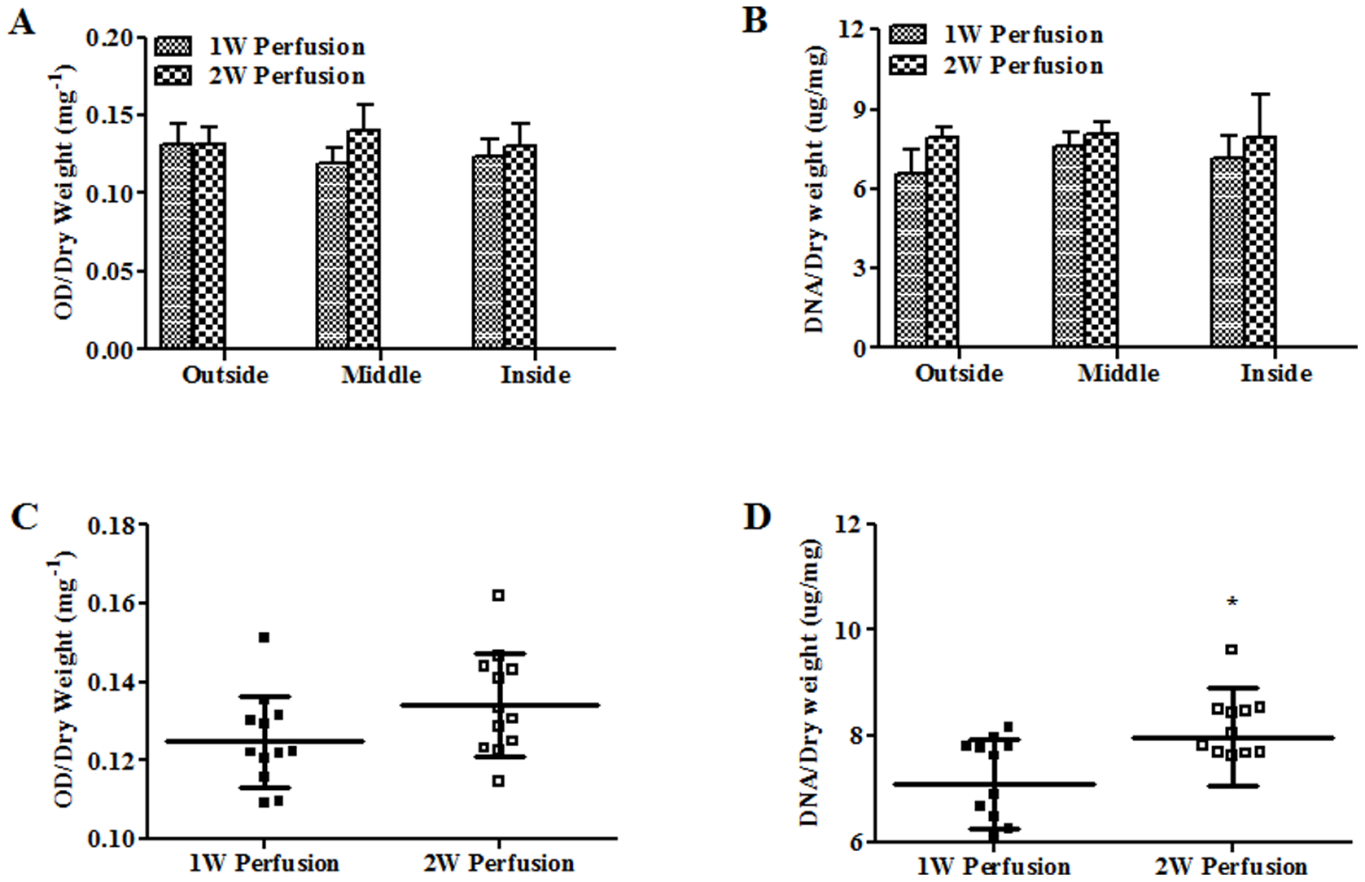
Metabolic activity and DNA content in macrotissues. (**A**) Metabolic activity in different regions of macrotissues (“Outside”, “Middle” and “Inside” as defined in Fig. 1B) was determined using MTT assay (n = 4). (**B**) All 12 data points from MTT assay for three regions of macrotissues were pooled and cellular activity of macrotissues perfusion cultured for 1 week (1W Perfusion) and 2 weeks (2W Perfusion) was compared. Similarly, DNA contents of three regions of macrotissues were shown (**C**) and compared between macrotissues perfusion cultured for 1 week and 2 weeks (**D**). ^*^ indicates significance compared to 1W Perfusion.

**Figure 6:**
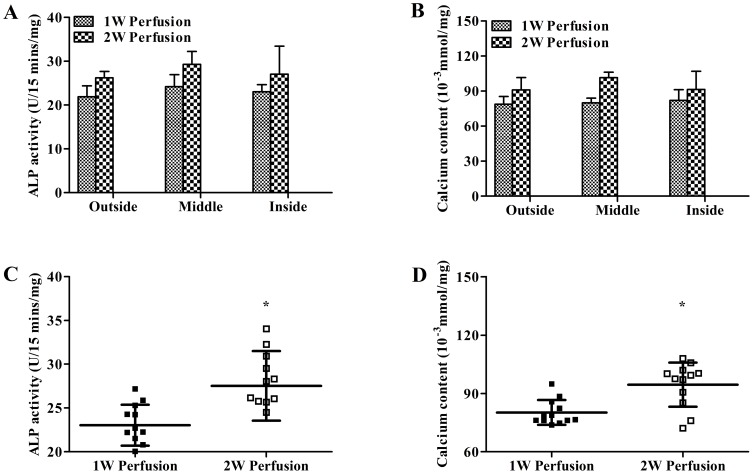
Quantification of ALP activity and calcium content in macrotissues. (**A**) ALP activity in different regions of macrotissues (“Outside”, “Middle” and “Inside” as defined in Fig. 1B) was determined (n = 4). (**B**) All 12 data points from three regions of macrotissues were pooled and ALP activity was compared between macrotissues perfusion cultured for 1 week (1W Perfusion) and 2 weeks (2W Perfusion). Similarly, calcium contents of three regions of macrotissues were shown (**C**) and compared between macrotissues perfusion cultured for 1 week and 2 weeks (**D**). ^*^ indicates significance compared to 1W Perfusion.

Macrotissues were also sampled from different regions as defined in Fig. 1B (“Inside”, “Middle”, and “Outside”, 4 samples for each region, 12 data points in total for each macrotissue) for biochemical assays. Based on MTT assay, no difference in metabolic activity was found among different regions in both macrotissues and between two macrotissues for the same regions (Fig. 5A). In addition, when 12 data points for each macrotissue were pooled and compared between two macrotissues, no difference was detected (Fig. 5C). No variations were seen in DNA content among different regions in both macrotissues and between two macrotissues for the same regions (Fig. 5B). However, when pooled data were compared, significantly higher DNA content was noted in the macrotissue perfused for 2 weeks (Fig. 5D). There was no difference for ALP activity and calcium content among different regions in both macrotissues and between the two macrotissues for the same regions (Fig. 6A, B). Notably, pooled data showed higher ALP activity and calcium content in the macrotissue perfused for 2 weeks (Fig. 6C, D).

### Ectopic osteogenesis in nude mice

**Figure 7:**
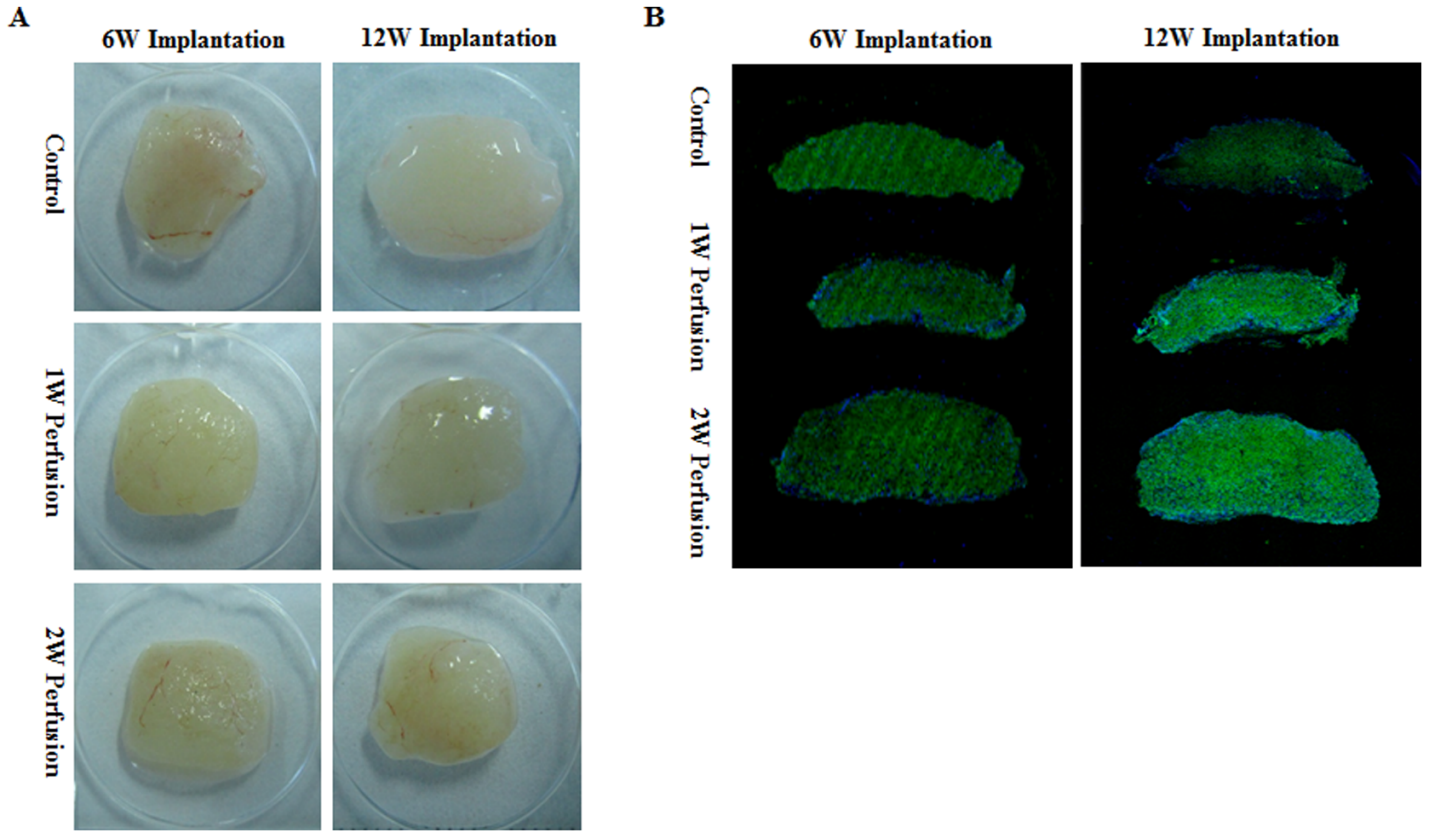
Gross view and cell viability of explants. Macrotissues (1W Perfusion and 2W Perfusion) were cut into 5 mm×10 mm×10 mm and subcutaneously implanted in nude mice for 6 weeks (6W Implantation) or 12 weeks (12W Implantation). As a control, same amount of microtissues without perfusion assembling was also directly implanted (Control). (**A**) Gross views of explants were shown. (**B**) Explants were evaluated with live/dead staining for cell viability.

One eighth of the macrotissues was implanted in each subcutaneous pocket of nude mice as illustrated in Fig. 1A. All explants had an integral structure with a whitish translucent appearance for both 6- and 12-week implantation (Fig. 7A). It was worth noting that, in “Control”, microtissues could be directly assembled into macrotissues upon implantation without *in vitro* perfusion assembling. Blood perfused vessels were noticed in all explants. For all three groups (“Control”, “1W Perfusion” and “2W Perfusion” in Fig. 7A), there was no obvious difference in gross view between the explants implanted for 6 and 12 weeks. Viable cells were present in all explants based on live/dead staining and no clear difference was noticed (Fig. 7B). However, with extended perfusion culture time (0, 1 and 2 weeks for “Control”, “1W Perfusion” and “2W Perfusion”, respectively), the explants were thicker, suggesting better mechanical strength to maintain the original shape in subcutaneous pockets.

**Figure 8:**
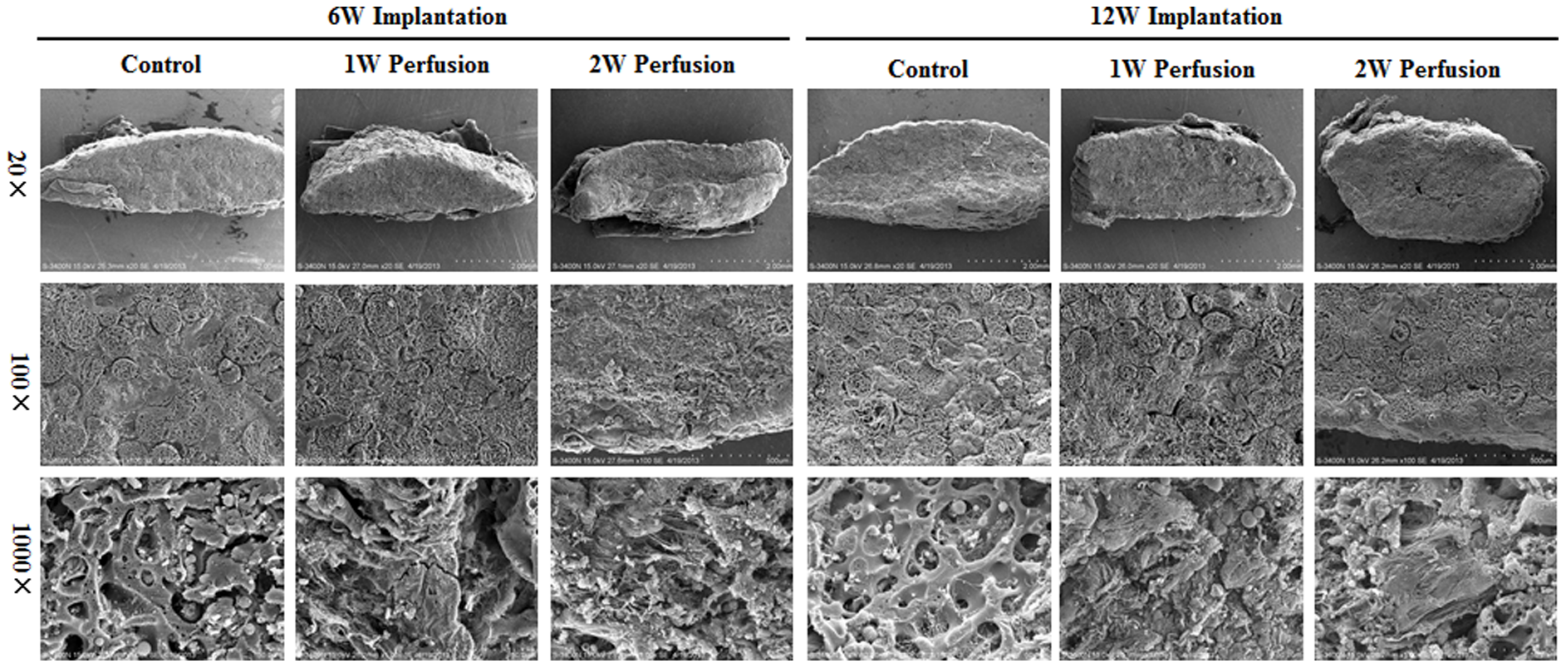
Microstructure of explants. Explants were cryofractured and subjected to SEM observation under magnifications of 20×, 100× and 1000×.

The microstructural features were examined using SEM (Fig. 8). While all explants displayed similar tight contextures at a magnification of 20, microstructural differences were noticed at higher magnifications. At 100×, cells filled in the spaces between microcarriers in all explants and the contour of microcarriers became invisible in the explants of 2-week perfused macrotissues. At 1000×, the internal porous structure of CultiSpher S microcarriers could be seen with very few minerals deposited in the explants of “Control”. In contrast, the characteristic structure of microcarriers was hardly discernible and mainly replaced with minerals and cells in the explants of perfused macrotissues. Nonetheless, no obvious structural difference was found between the explants implanted for 6 and 12 weeks.

**Figure 9:**
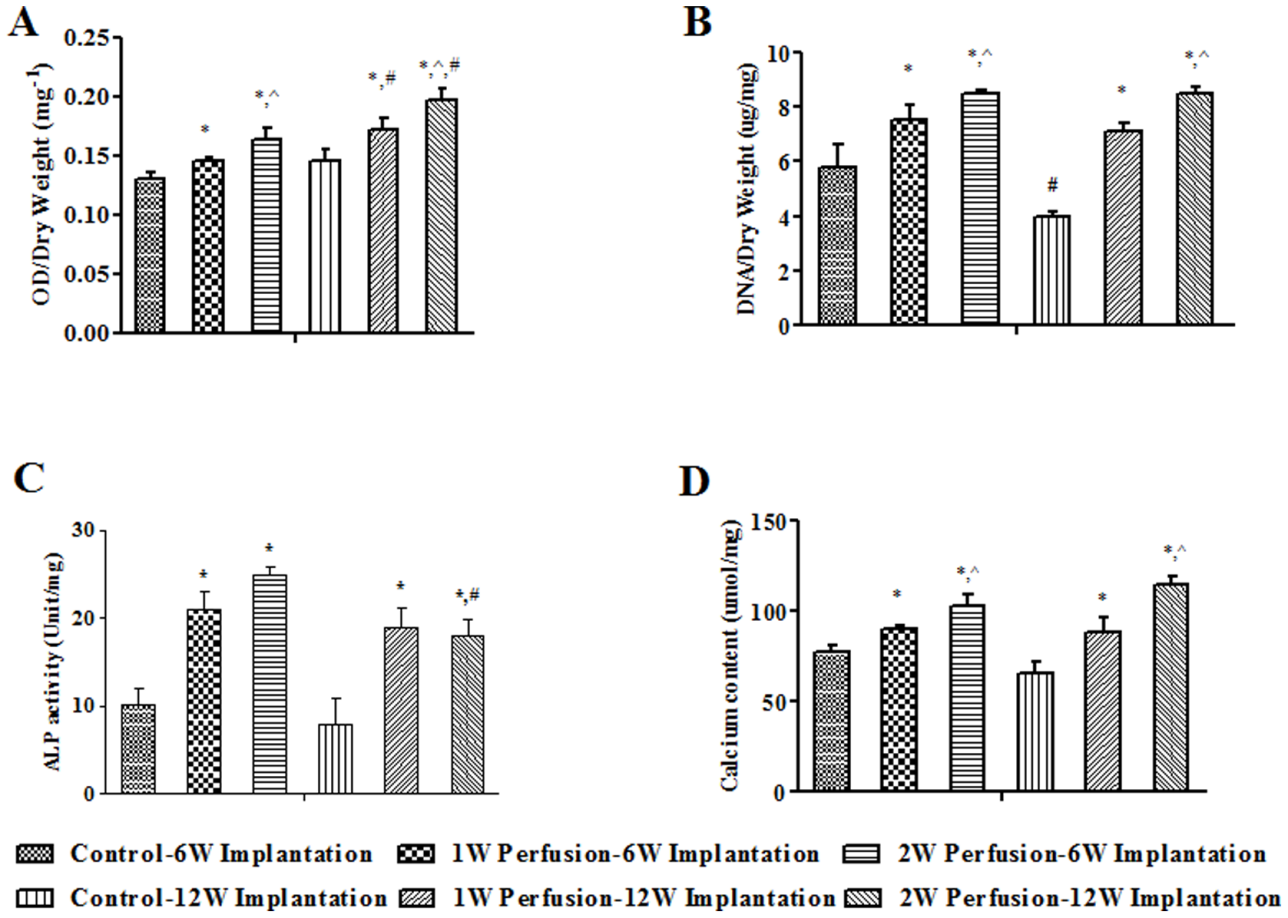
Biochemical analysis of explants. Metabolic activity (**A**), DNA content (**B**), ALP activity (**C**) and calcium content (**D**) in explants were quantified (n = 4). Control-6W Implantation and Control-12W Implantation: non-assembled microtissues were implanted for 6 and 12 weeks, respectively; 1W Perfusion-6W Implantation and 1W Perfusion-12W Implantation: macrotissues perfused for 1 week were implanted for 6 and 12 weeks, respectively; 2W Perfusion-6W Implantation and 1W Perfusion-12W Implantation: macrotissues perfused for 2 weeks were implanted for 6 and 12 weeks, respectively. ^*^ indicates significance compared to respective control; ^∧^ indicates significance compared to respective 1W Perfusion; ^#^ indicates significance compared to respective 6W Implantation.

As shown in Fig. 9A, with prolonged *in vitro* perfusion culture time, the corresponding explants showed significantly higher metabolic activity regardless of implantation time based on MTT assay. In addition, for the explants of 1- and 2-week perfused macrotissues, implantation for 12 weeks enhanced metabolic activity over that for 6 weeks. DNA content in explants also increased with extended culture time (Fig. 9B). However, there was no difference between the explants implanted for 6 and 12 weeks, except a lower DNA content in “Control” after 12-week implantation. With the same implantation time, explants of both perfused macrotissues had similar ALP activities, which were higher than that of “Control” (Fig. 9C). It was noted that ALP activity of explants of 2-week perfused macrotissue after 12-week implantation was slightly lower than that after 6-week implantation. Calcium content in explants was higher with *in vitro* longer culture time and no difference was detected between the explants after 6- and 12-week implantation (Fig. 9D).

**Figure 10:**
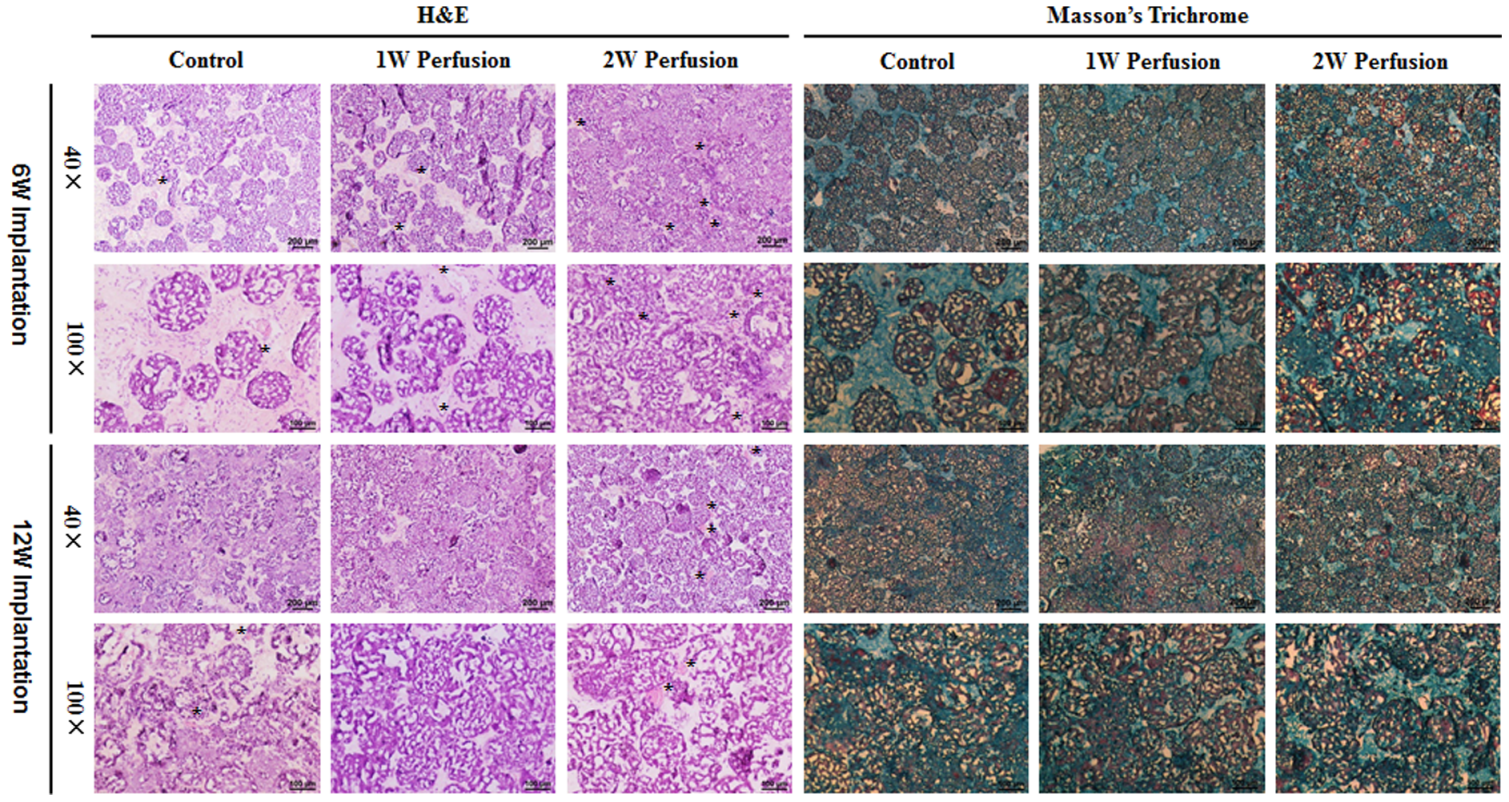
Histological analysis of explants. Sections of the explants were subjected to H&E staining and Masson's trichrome staining. Images were acquired under magnifications of 40× and 100×. * indicates the presence of blood vessel. Green color: mineralized bone matrix.

Based on H&E staining, explants after 6-week implantation of the macrotissues with longer culture time had much denser contexture and after 12-week implantation, it became similar (Fig. 10). However, in all explants, ECM filled in all the spaces between microcarriers. While cells were confined to the spaces between microcarriers for the explants of “Control” after 6-week implantation, the inner space of microcarriers in all other explants were infiltrated with cells. Breakage of microcarriers, which indicated the degradation, was noticed after a shorter implantation time (6 weeks) in explants of 2-week perfused macrotissues. According to Masson's trichrome staining, significant amounts of mineralized collagen were found in all explants and explants of 2-week perfused macrotissues had denser and more homogeneously distributed bone matrix than others. In general, 12-week implantation resulted in more mineralized matrix. However, these bone matrix seemed to be pretty immature. The immunohistochemical staining demonstrated the higher expression of collagen I and osteocalcin in explants of 2-week perfused macrotissues after 6-week implantation (Fig. S2). Micro-CT analysis showed that a few bone nodules in explants, with the highest in those of 2-week perfused macrotissues, and no difference was found between the explants after 6- and 12-week implantation (Fig. S3).

**Figure 11:**
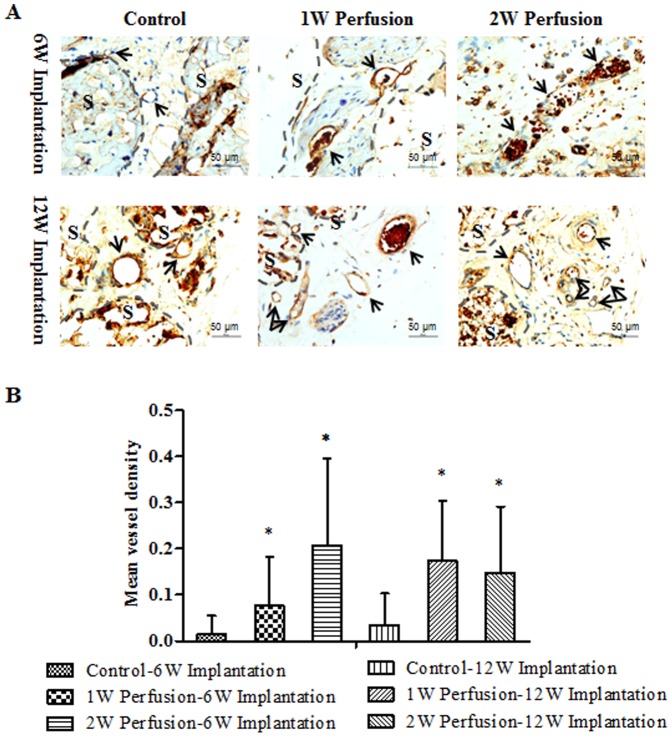
Immunohistostaining of CD31. (A) Representative images of stained sections of the explants under the magnification of 400×; (B) the mean vessel density quantified with Image J software. “S” indicates microcarriers; arrow indicates blood perfused vessel; ^*^ indicates significance compared to respective control.

Based on H&E staining, the infiltration of blood vessels could been seen in all explants. There tended to have more blood vessels in the explants of the macrotissues perfused for 2 weeks and no difference in blood vessel density between the explants after 6- and 12-week implantation for all three groups. To further characterize the vascularization, the expression of CD31 was determined by immunohistostaining. As shown in Fig. 11A, positive staining was observed in all explants, confirming the infiltration of blood vessels. Based on a quantitative analysis of the positive staining (mean vessel density), it was shown that the vessel density was significantly higher in the explants of perfused macrotissues (Fig. 11B). However, no difference was detected between the explants of 1-week and 2 week perfused macrotissues.

## Discussion

In our previous report, a modular approach was established to fabricate centimeter-sized bone tissue [21]. However, how *in vitro* perfusion culture time would affect tissue properties and how the osteogenesis of *in vitro* assembled tissues would be *in vivo* have not been evaluated. This is critical concerning future translation of this modular approach into clinical settings. In the present study, microtissues were prepared by following the previously described protocol [21]. Microtissues were then assembled in perfusion culture for 1 or 2 weeks and macrotissues were implanted into subcutaneous pockets of nude mice for 6 or 12 weeks to evaluate ectopic osteogenesis.

Within 28 days, spheroidal microtissues of 700∼800 µm in diameter with high cell viability and osteogenic differentiation were fabricated in spinner flasks. This was a continuous process including cell seeding, proliferation and osteogenic differentiation and readily for scaling-up. Considering bone tissue engineering that generally needs a large volume of tissue replacements, it is advantageous to develop a method being readily scaled up for tissue fabrication [24]. It should be noted that spontaneous aggregation of hAMSCs-laden CultiSpher S microcarriers in spinner flasks is common, which suggests an intrinsic mechanism of assembling for cell-laden microcarriers [13]. It has been suggested that the aggregation process is cell- and microcarrier-type dependent, possibly relying on cell proliferation, migration, ECM production and microcarrier property [25]–[27]. In addition, by controlling culture condition, aggregates within an appropriate size can be made to ensure high cell viability [15].

In perfusion culture, microtissues readily assembled into integral macrotissues displaying bone tissue characteristics within both 1 and 2 weeks. Compared to those after 1-week culture, the assembled tissues had much tighter contexture after 2-week culture. Inside macrotissues, the space between cell-laden microcarriers were filled with cells and ECM, especially after 2-week perfusion, possibly leading to inefficient interstitial fluid flow perfusion. This was different from the study by Khan et al., wherein cell-laden microcarriers were only loosely packed, thus forming interconnected microchannels for *in vitro* perfusion [28]. Despite of this concern, slightly higher DNA content, ALP activity and calcium deposition in macrotissues were seen after 2-week culture than 1-week culture, which suggested that a longer culture time supported better tissue formation and nutrition deprivation was not encountered in the macrotissues. Indeed, Twal et al. also observed that the mechanical properties of tubular tissue constructs prepared from cell-laden CultiSpher G were improved along with extended culture time within 17 days in static culture [29]. In contrast, in our previous study, inside a macrotissue assembled from HFs-laden Cytopore-2 microcarriers (made of non-degradable crosslinked cotton cellulose), significant cell death was observed after perfusion culture for 2 weeks, which was attributed to a mass transport limit due to the formation of a very dense layer of cells at the periphery of the macrotissue [23]. Hence, an optimal *in vitro* culture time should be selected for assembling macrotissues, which might be dependent on types of both cells and microcarriers. In line with this, Imparato et al. demonstrated that the nature of microcarriers (cross-linking density) played critical roles in dictating the tissue properties of HFs-laden gelatin microcarriers-assembled macrotissues during a 6-week culture period [30].

So far, *in vivo* performance of assembled macrotissues following a modular approach has not been reported yet. Although David et al. subcutaneously implanted pooled perfusion cultured cubic coral scaffolds (3 mm×3 mm×3 mm) seeded with MSCs in sheep, these constructs were only weakly associated with each other [31]. In the present study, microtissues made of hAMSCs-laden CultiSpher S microcarriers were assembled into an integral structure *in vitro* and then further developed into a tissue construct showing very tight contexture *in vivo*. Upon subcutaneous implantation, the tissues could maintain cell metabolic activity and osteogenesis and however, longer implantation time did not lead to higher ALP activity and calcium deposition. Several studies have evidenced that implanted cells in scaffolds can only survive within several weeks [32], [33]. In the present study, it was not clear whether the metabolic activity was contributed by hAMSCs or infiltrated host cells. Notably, directly implanted microtissues could also assemble into an integral macrotissue. As a matter of fact, injectable cell-laden microcarriers have been shown promising for tissue repair [34], [35]. But, compared to those explants of perfuse-cultured macrotissues, directly implanted microtissues were inferior in cell metabolic activity and osteogenesis.

Depending on the duration of *in vitro* perfusion culture (0, 1 and 2 weeks), the explants displayed different microstructural features, with longer *in vitro* culture time showing more profound remodeling. In consistence, degradation of microcarriers was most pronounced in explants of 2-week perfused macrotissues. While longer implantation time (12 weeks) did not seem to change both ALP activity, calcium content as well as microstructures under SEM in explants, degradation of microcarriers tended to be more extensive based on histological analysis. Huss et al. reported the degradation of CultiSpher S microcarriers after intradermally implanted in human [36]. This is advantageous since a biodegradable scaffold should facilitate cellular remodeling during tissue morphogenesis [30]. Notably, in the present study, degradation of microcarriers was possibly associated with the elaboration of mineralized collagen matrix in the inner spaces of microcarriers as for the explants of 2-week perfused macrotissues.

More perfusable blood vessels were present in explants of perfused macrotissues after both 6- and 12 week implantation, which should be beneficial for long-term survival of implanted tissues. Direct subcutaneous implantation of cell-laden microcarriers had previously been reported to solicit infiltration of blood vessels [37]. It has been implicated in several studies that infiltration of blood vessels is critical for bone tissue regeneration in implanted cells/scaffolds constructs [32], [33]. The present study suggested that *in vitro* culture permitted better maturation of tissues, which could promote implants/host interaction, leading to more efficient establishment of a vascular network.

Bone formation was further characterized with Masson's trichrome staining and immunohistology, confirming the presence of mineralized bone matrix in all explants. This suggested that *in vitro* osteogenically differentiated hAMSC-laden CultiSpher S microcarriers well maintained the osteogenesis *in vivo* without the presence of osteogenic induction factors. Moreover, the explants of 2-week perfused macrotissues had more mineralized matrix with more homogenous spatial distribution. This was potentially attributed to both degradation of microcarriers and blood vessel infiltration. However, although longer implantation (12 weeks versus 6 weeks) seemed to have intensified bone matrix deposition, bone tissue still tended to be immature after 12-week implantation in all explants, which could be attributed to several factors. An ectopic subcutaneous implantation model might be inefficient in inducing bone tissue maturation [33], [38], [39]. In addition, osteogenic differentiation capacity of hAMSCs might be relatively inefficient in comparison with MSCs derived from other sources such as bone marrow [40].

In conclusion, a spinner flask-based culture method was efficient in fabricating microtissues with bone characteristics using hAMSCs-laden CultiSpher S microcarriers. Microtissue assembling was robust in perfusion culture and tissue property was dependent on perfusion time. Subcutaneous implantation of assembled macrotissues was able to maintain bone characteristics, showing active cellular remodeling, infiltration of blood vessels and deposition of mineralized bone matrix, which was correlated with *in vitro* perfusion culture time. This study demonstrates that a modular approach is promising for fabricating viable tissue replacements for bone regeneration.

## Supporting Information

Figure S1 **The setup of perfusion culture system.** (A) The system was composed of three functional parts: perfusion chamber, peristaltic pump and medium reservoir; (B) The glass cylindrical perfusion chamber; (C) The assembled macrotissue sandwiched between two perforated plastic gaskets.
(DOCX)

Figure S2 **Immunohistological analysis of collagen I and osteocalcin.** Sample sections were incubated with primary antibodies against collagen I (1∶200; Rabbit polyclonal antibody against human Collagen I aa 1–1464, Abcam) or osteocalcin (1∶100; Clone 7D1.13, Mouse monoclonal antibody against KLH-conjugated linear peptide corresponding to Human Osteocalcin, Millipore), followed by horseradish peroxidase (HRP)-conjugated anti-rabbit or anti-mouse secondary antibody (1∶200 in PBS containing 1% BSA, Santa Cruz) and color development with diaminobenzidine tetrahydrochloride (Santa Cruz). Nuclei were counterstained with hematoxylin. The whole sample sections were imaged sequentially and photos were compiled to show the whole sections. Scale bar: 500 µm.
(DOCX)

Figure S3 **The micro-CT analysis of explants.**
(DOCX)
