# Supplementary material for: Ectopic Osteogenesis of Macroscopic Tissue Constructs Assembled from Human Mesenchymal Stem Cell-Laden Microcarriers through In Vitro Perfusion Culture
Source: PLoS One. 2014 Oct 2;9(10):e109214. doi: 10.1371/journal.pone.0109214 (PMC4183582; doi:10.1371/journal.pone.0109214)
Supplement: Figure S1 — The setup of perfusion culture system. (A) The system was composed of three functional parts: perfusion chamber, peristaltic pump and medium reservoir; (B) The glass cylindrical perfusion chamber; (C) The assembled macrotissue sandwiched between two perforated plastic gaskets. (DOCX) [file pone.0109214.s001.docx]

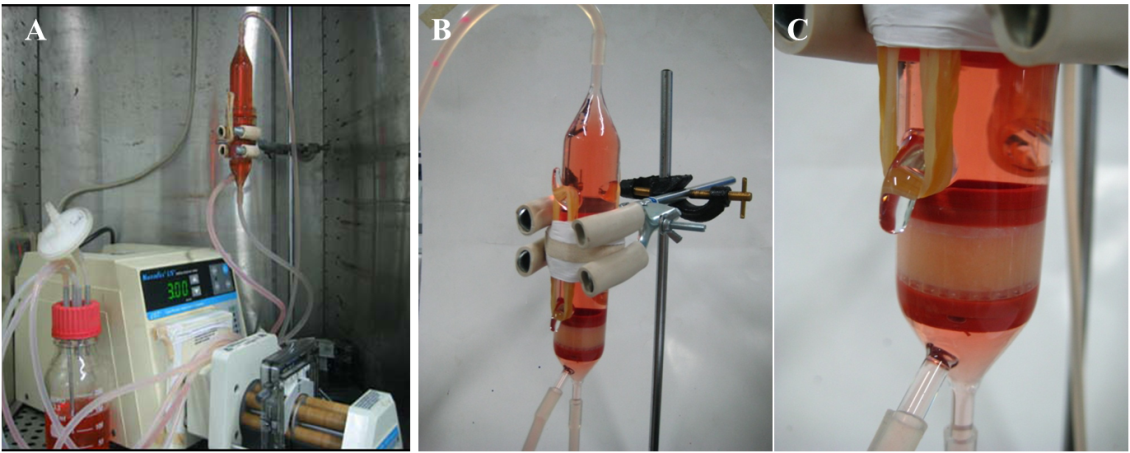


**Figure S1. The setup of perfusion culture system.** (A) The system was composed of three functional parts: perfusion chamber, peristaltic pump and medium reservoir; (B) The glass cylindrical perfusion chamber; (C) The assembled macrotissue sandwiched between two perforated plastic gaskets.
