# Supplementary material for: Ectopic Osteogenesis of Macroscopic Tissue Constructs Assembled from Human Mesenchymal Stem Cell-Laden Microcarriers through In Vitro Perfusion Culture
Source: PLoS One. 2014 Oct 2;9(10):e109214. doi: 10.1371/journal.pone.0109214 (PMC4183582; doi:10.1371/journal.pone.0109214)
Supplement: Figure S2 — Immunohistological analysis of collagen I and osteocalcin. Sample sections were incubated with primary antibodies against collagen I (1∶200; Rabbit polyclonal antibody against human Collagen I aa 1–1464, Abcam) or osteocalcin (1∶100; Clone 7D1.13, Mouse monoclonal antibody against KLH-conjugated linear peptide corresponding to Human Osteocalcin, Millipore), followed by horseradish peroxidase (HRP)-conjugated anti-rabbit or anti-mouse secondary antibody (1∶200 in PBS containing 1% BSA, Santa Cruz) and color development with diaminobenzidine tetrahydrochloride (Santa Cruz). Nuclei were counterstained with hematoxylin. The whole sample sections were imaged sequentially and photos were compiled to show the whole sections. Scale bar: 500 µm. (DOCX) [file pone.0109214.s002.docx]

**
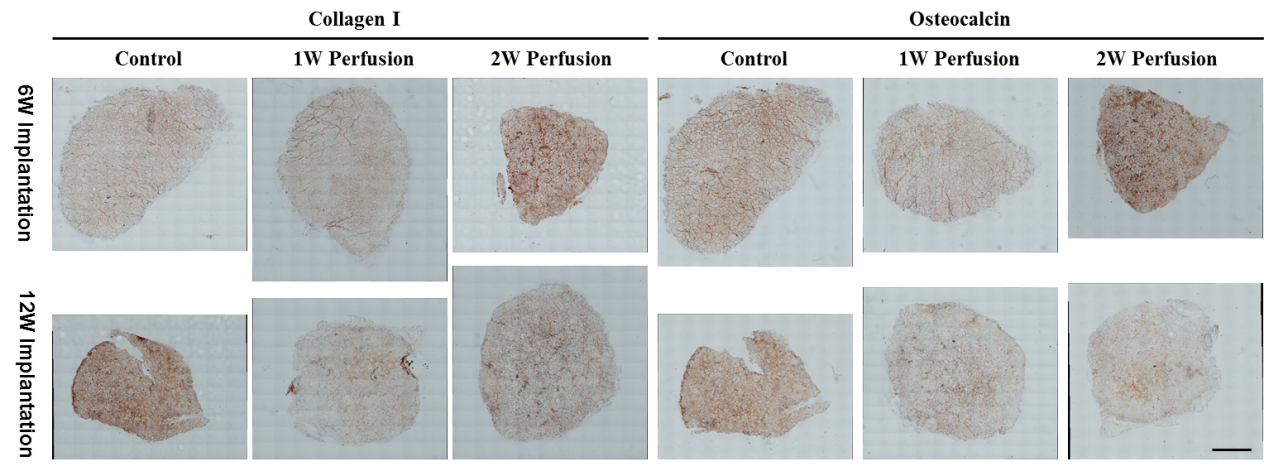
Figure S2.** **Immunohistological analysis of collagen I and osteocalcin.** Sample sections were incubated with primary antibodies against collagen I (1:200; Rabbit polyclonal antibody against human Collagen I aa 1-1464, Abcam) or osteocalcin (1:100; Clone 7D1.13, Mouse monoclonal antibody against KLH-conjugated linear peptide corresponding to Human Osteocalcin, Millipore), followed by horseradish peroxidase (HRP)-conjugated anti-rabbit or anti-mouse secondary antibody (1:200 in PBS containing 1% BSA, Santa Cruz) and color development with diaminobenzidine tetrahydrochloride (DAB, Santa Cruz). Nuclei were counterstained with hematoxylin. The whole sample sections were imaged sequentially and photos were compiled to show the whole sections. Scale bar: 500 μm.
