# Supplementary figures and images for: Ectopic Osteogenesis of Macroscopic Tissue Constructs Assembled from Human Mesenchymal Stem Cell-Laden Microcarriers through In Vitro Perfusion Culture
Source: PLoS One. 2014 Oct 2;9(10):e109214. doi: 10.1371/journal.pone.0109214 (PMC4183582; doi:10.1371/journal.pone.0109214)

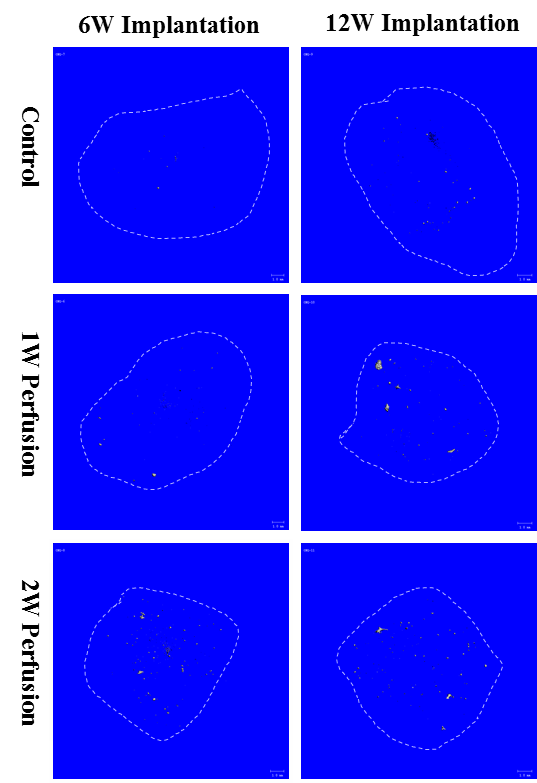


**Figure S3. The micro-CT analysis of explants.**

Supplement: Figure S3 — The micro-CT analysis of explants. (DOCX) [file pone.0109214.s003.docx]
